# Sex Differences in Aging-related Myocardial Stiffening Quantitatively Measured with MR Elastography

**DOI:** 10.1148/ryct.230140

**Published:** 2024-05-23

**Authors:** Arvin Arani, Matthew C. Murphy, Huzefa Bhopalwala, Shivaram P. Arunachalam, Phillip J. Rossman, Joshua D. Trzasko, Kevin Glaser, Yi Sui, Tina Gunderson, Adelaide M. Arruda-Olson, Armando Manduca, Kejal Kantarci, Richard L. Ehman, Philip A. Araoz

**Affiliations:** From the Departments of Radiology (A.A., M.C.M., H.B., S.P.A., P.J.R., J.D.T., K.G., Y.S., A.M., K.K., R.L.E., P.A.A.), Quantitative Health Science (T.G.), and Cardiology (A.M.A.O.), Mayo Clinic, 200 First St SW, Rochester, MN 55905.

**Keywords:** Cardiac, Elastography, Biological Effects, Experimental Investigations, Sexual Dimorphisms, MR Elastography, Myocardial Shear Stiffness, Quantitative Stiffness Imaging, Aging Heart, Myocardial Biomechanics, Cardiac MRE

## Abstract

**Purpose:**

To investigate the feasibility of using quantitative MR elastography (MRE) to characterize the influence of aging and sex on left ventricular (LV) shear stiffness.

**Materials and Methods:**

In this prospective study, LV myocardial shear stiffness was measured in 109 healthy volunteers (age range: 18–84 years; mean age, 40 years ± 18 [SD]; 57 women, 52 men) enrolled between November 2018 and September 2019, using a 5-minute MRE acquisition added to a clinical MRI protocol. Linear regression models were used to estimate the association of cardiac MRI and MRE characteristics with age and sex; models were also fit to assess potential age-sex interaction.

**Results:**

Myocardial shear stiffness significantly increased with age in female (age slope = 0.03 kPa/year ± 0.01, *P* = .009) but not male (age slope = 0.008 kPa/year ± 0.009, *P* = .38) volunteers. LV ejection fraction (LVEF) increased significantly with age in female volunteers (0.23% ± 0.08 per year, *P* = .005). LV end-systolic volume (LVESV) decreased with age in female volunteers (−0.20 mL/m^2^ ± 0.07, *P* = .003). MRI parameters, including T1, strain, and LV mass, did not demonstrate this interaction (*P* > .05). Myocardial shear stiffness was not significantly correlated with LVEF, LV stroke volume, body mass index, or any MRI strain metrics (*P* > .05) but showed significant correlations with LV end-diastolic volume/body surface area (BSA) (slope = −3 kPa/mL/m^2^ ± 1, *P* = .004, *r*^2^ = 0.08) and LVESV/BSA (−1.6 kPa/mL/m^2^ ± 0.5, *P* = .003, *r*^2^ = 0.08).

**Conclusion:**

This study demonstrates that female, but not male, individuals experience disproportionate LV stiffening with natural aging, and these changes can be noninvasively measured with MRE.

**Keywords:** Cardiac, Elastography, Biological Effects, Experimental Investigations, Sexual Dimorphisms, MR Elastography, Myocardial Shear Stiffness, Quantitative Stiffness Imaging, Aging Heart, Myocardial Biomechanics, Cardiac MRE

*Supplemental material is available for this article*.

Published under a CC BY 4.0 license.

## Graphical abstract

**Figure:**
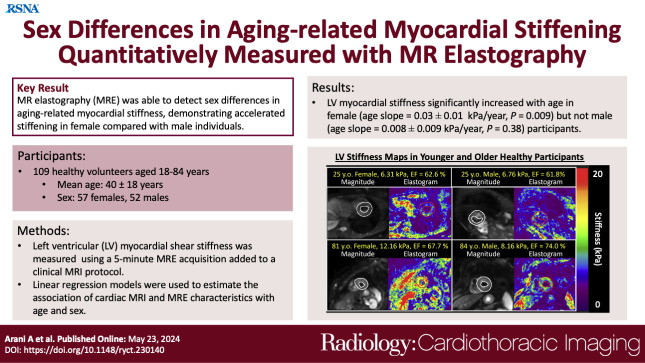

**Summary**

The use of MR elastography to detect sex-related differences in myocardial shear stiffness was feasible and suggested that female individuals may experience accelerated stiffening compared with male individuals as they age.

**Key Points**

■ Quantitative MR elastography was used to noninvasively measure myocardial stiffness in 109 healthy participants in the 18–84-year age range.
■ Left ventricular myocardial shear stiffness significantly increased with age in female (age slope = 0.03 kPa/year ± 0.01, *P* = .009) but not male (age slope = 0.008 kPa/year ± 0.009, *P* = .38) participants.
■ This feasibility study demonstrates that female individuals may be more prone to myocardial stiffening than male individuals during natural aging.

## Introduction

Elevated myocardial stiffness is both a precursor and a biologic response to a spectrum of cardiovascular diseases that account for one in five deaths in the United States annually (1), even in the COVID-19 era. Stiffness is a long sought-after metric due to its impact on cardiac function. Elevated myocardial stiffness causes poor function and restrictive diastolic filling that can lead to heart failure, even with a normal left ventricular (LV) ejection fraction (LVEF) (2). However, the invasiveness of existing techniques for measuring myocardial stiffness in vivo, which include catheter pressure volume measurements and myocardial biopsies, hampers the widespread application of myocardial stiffness as a practical clinical biomarker and makes measurements in healthy individuals challenging.

Shear wave elastography is an emerging imaging approach for measuring myocardial shear stiffness in vivo (3–8). Shear wave elastography approaches rely on external or intrinsic vibrating sources to generate shear waves inside a tissue of interest. An imaging technique, either MRI or US, then measures the vibrational displacements in the tissue (3,6). The displacement field is used to calculate a shear stiffness map through one of several mathematical techniques collectively referred to as *inversion algorithms* (9). Thus, MR elastography (MRE) can provide a noninvasive quantitative measure of stiffness.

Heart failure hospitalization rates by sex show disproportionate increases in aging women (10). Heart failure with preserved ejection fraction is twice as prevalent in female than in male individuals (11), and limited treatment options have contributed to improved overall survival in male but not in female individuals (12). Furthermore, global and regional quantitative myocardial shear stiffness changes during menopausal transition are currently unknown and could be an essential mechanism for combating the sex disparities in patients with heart failure. Menopause has been associated cross-sectionally with worse diastolic function and longitudinally with adverse LV and left atrial remodeling (13). Hypertension is more prevalent in female individuals than in male individuals with heart failure, increasing heart failure risk by threefold in female individuals and only twofold in male individuals (14). Undetected changes in myocardial shear stiffness, due to menopause for example, could be a major contributor to these trends.

Currently, a three-dimensional (3D) high-frequency cardiac MRE technique is being developed that has shown a high level of concordance with dynamic material testing (15), is feasible in healthy individuals (16), and has the sensitivity to measure elevated myocardial shear stiffness in patients with cardiac amyloidosis (17). This study aimed to evaluate the feasibility of cardiac MRE in measuring aging- and sex-related differences in LV myocardial shear stiffness.

## Materials and Methods

Four of the authors (K.G., M.C.M., A.M., and R.L.E.) and the Mayo Clinic have intellectual property rights and potential financial interest in some of the MRE technology (active and passive drivers, pulse sequences, and inversions) used in this study. The study data were analyzed and controlled by two authors (A.A. and T.G.) who did not have this potential conflict of interest.

### Study Participants

In this prospective study, LV systolic myocardial shear stiffness was measured in 109 healthy volunteers (age range: 18–84 years) from the general population of 5.7 million in Minnesota, using MRE. Recruitment started in November 2018 and ended in September 2019. This Health Insurance Portability and Accountability Act–compliant study was approved by our institutional review board, and written informed consent was received from each participant prior to study enrollment. The volunteers had no history of coronary artery disease, heart failure, hypertension, hypertensive medication, valvular heart disease, congenital heart disease, or diabetes. The volunteers also had no symptoms of chest pain or shortness of breath and exhibited normal heart sounds and breath sounds. Individuals were excluded if they had contraindications to MRI scanning.

### MRE Acquisition

**Figure 1:**
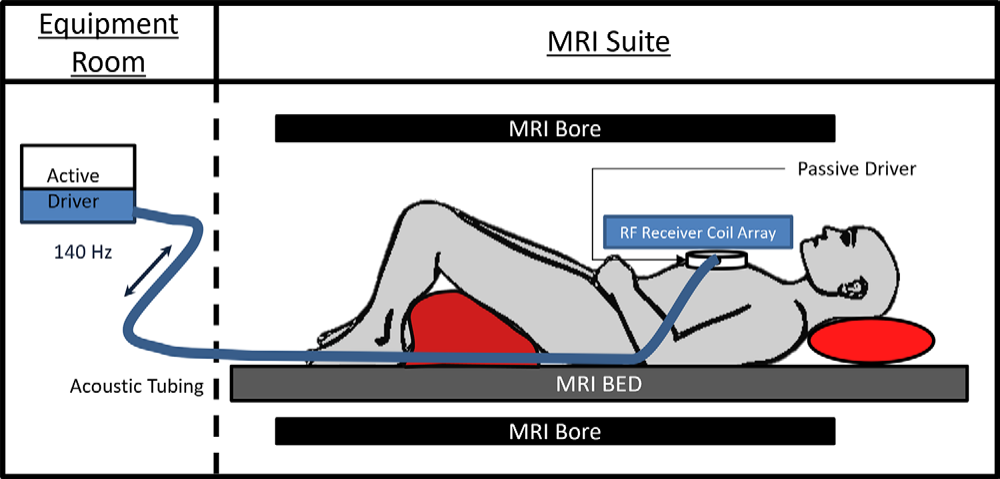
Cardiac MR elastography experimental setup. A participant is placed supine feet first into the bore. An active driver delivers 140-Hz vibrations through acoustic tubing to a passive cardiac driver that is strapped to the individual’s chest. Shear waves are transmitted into the myocardium, and the wave displacement field is imaged with MRI. RF = radiofrequency.

Cardiac MRE was used to quantitatively measure myocardial shear stiffness across the LV of each participant. The experimental setup is shown in Figure 1. An active driver, outside of the scan room, generated acoustic vibrations that were transmitted through acoustic tubing to a passive driver in contact with the participant. The passive driver’s surface was coated with acoustic gel and placed in direct contact with the participant’s skin, just to the left of the sternum and superior to the xiphoid process. An elastic tension strap was used to keep the passive driver in place and to help improve coupling between the passive driver diaphragm and the participant’s skin surface. To help avoid signal interference from shear wave vibrations, cardiac gating was performed by placing electrocardiographic (ECG) leads on the back of the left shoulder of each participant instead of on the chest.

Imaging was performed with a 1.5-T closed-bore MR imager (Optima MR450w; GE HealthCare) in an oblique orientation to obtain short-axis MRE images of the heart using the built-in, receive-only, integrated whole-body phased array system (GEM anterior-posterior array; GE HealthCare). Imaging was conducted using the same procedure as previously described (17). Briefly, a flow-compensated, cardiac-gated, spin-echo, single-shot echo-planar imaging MRE sequence was used with the following parameters: repetition time matched to each volunteer’s heart rate with ECG gating; echo time = 69 msec; field of view = 32 cm; 64 × 64 image matrix; parallel imaging acceleration factor = 2; five contiguous 5-mm-thick axial sections; two motion-encoding gradient pairs on each side of the refocusing pulse matched to the vibration frequency; alternating x, y, z, and 0 motion-encoding gradient directions; and four phase offsets spaced evenly over one vibration period, at a vibration frequency of 140 Hz. For image processing purposes, the image matrix was reformatted to 256 × 256 × 20 to give an isotropic voxel size of 1.25 mm. Images were acquired at the minimum delay possible in the cardiac cycle (approximately 100 msec) after the R-wave ECG trigger, which is believed to be the most reproducible phase in the cardiac cycle. Sixteen volunteers underwent both full-field-of-view and the previously described reduced-field-of-view (18) cardiac MRE within the same examination. These data were used to calculate the reproducibility of our shear stiffness estimates by calculating the intraclass correlation coefficient (19) and concordance coefficient (20).

### MRE Postprocessing and Inversion

MRE inversion was implemented by taking the curl of the 3D displacement field and employing a 3D local frequency estimation algorithm (21) to invert the wave field and generate shear stiffness maps. This algorithm was chosen because it is very robust in the presence of noise and is not affected by wave reflections. The LV of the heart was semiautomatically segmented using commercial segmentation software (Cvi42 version 5.16.2; Circle Cardiovascular Imaging), and octahedral shear strain signal-to-noise ratio (OSS-SNR) (22) was calculated on the curl wave fields. The LV mask was eroded by two pixels in all directions to reduce edge effects. The median shear stiffness and mean OSS-SNR over the remaining volume were measured. An MRE examination was considered successful only if the mean OSS-SNR was greater than 1.17.

### Cardiac MRI Processing

For measurement of myocardial mass and volumes, cardiac MRI balanced steady-state free precession (bSSFP) images were obtained in the short axis. The following imaging parameters were used to acquire 15 sections with the bSSFP acquisition: a field of view of 38 cm, imaging matrix of 224 × 224, repetition time/echo time = 3.3 msec/1.1 msec, section thickness of 8 mm, flip angle of 60°, and a total scan time of approximately 2 minutes with 15 breath holds of approximately 17 seconds (depending on the heart rate). The short-axis bSSFP images were used to calculate LV mass, strain (peak global radial, peak global circumferential, and peak global longitudinal), and volumes using commercially available software (Cvi42) by manually tracing the LV endocardial and epicardial contours (performed by P.A.A., a cardiovascular radiologist with greater than 20 years of experience, in consensus with H.B., a cardiovascular radiology fellow with 1 year of experience). Both tracers were blinded to all MRE results.

Contrast-free native T1 mapping was conducted using a short-axis midsection modified Look-Locker inversion recovery (MOLLI) acquisition (23) with the following parameters: six inversion times of 200 msec, 280 msec, 360 msec, 1200 msec, 1280 msec, and 1360 msec; 38.0 cm (256 × 256 acquisition matrix) field of view; 10-mm section thickness; repetition time, 3500 msec; echo time, 1500 msec time; one signal average; and flip angle of 35°. The commercially available software package (Cvi42) was used to calculate the mean T1 measurements in the left and right ventricular myocardium (including the papillary muscles) with the MOLLI images.

### Statistical Analysis

Descriptive statistics were used to summarize participant characteristics. Linear regression models were used to estimate the association of cardiac MRI metrics with age and sex; models were also fit to assess potential age-sex interaction. Age was considered a continuous variable for all statistical models, unless otherwise indicated. Errors in all parameter estimates are reported as standard errors. Analyses were performed using R version 3.6.2 (R Foundation for Statistical Computing). To determine if cardiac MRE gave complementary information to existing MRI metrics, linear regression models, created using the MATLAB Statistical Toolbox (R2019b; MathWorks), were used to model the direct correlation of cardiac MRE–measured shear stiffness with T1, strain, and LVEF. As strain measurements are influenced by both the contractility of tissue as well as shear stiffness, the same linear regression model was used to compare each strain metric with LVEF (a measure of contractility) in addition to MRE-measured shear stiffness. F-statistics comparing the linear model to a constant model were used to determine significant differences. Nonlinear models were also tested but did not provide statistical improvements over linear models (data not shown). Also, a secondary analysis was performed that excluded volunteers younger than the age of 30 years to ensure nonuniform sampling distribution did not interfere with our findings. The same trends were observed in volunteers 30 years and older as compared with the full cohort (data not included); therefore, only a single cohort analysis was reported in this study. For all statistical comparisons, a *P* value of less than .05 was considered statistically significant.

## Results

### Participant Characteristics

The study included 109 healthy participants aged 18–84 years (mean age, 40 years ± 18 [SD]), with 57 women and 52 men. Participant demographics by age are shown in Table 1.

**Table 1:**
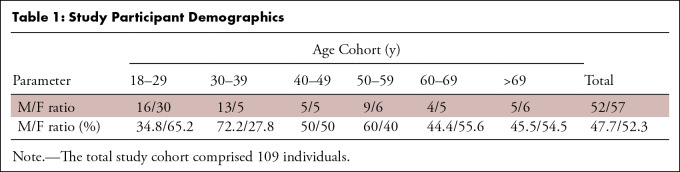
Study Participant Demographics

### MRE and MRI Age and Sex Model Results

**Figure 2:**
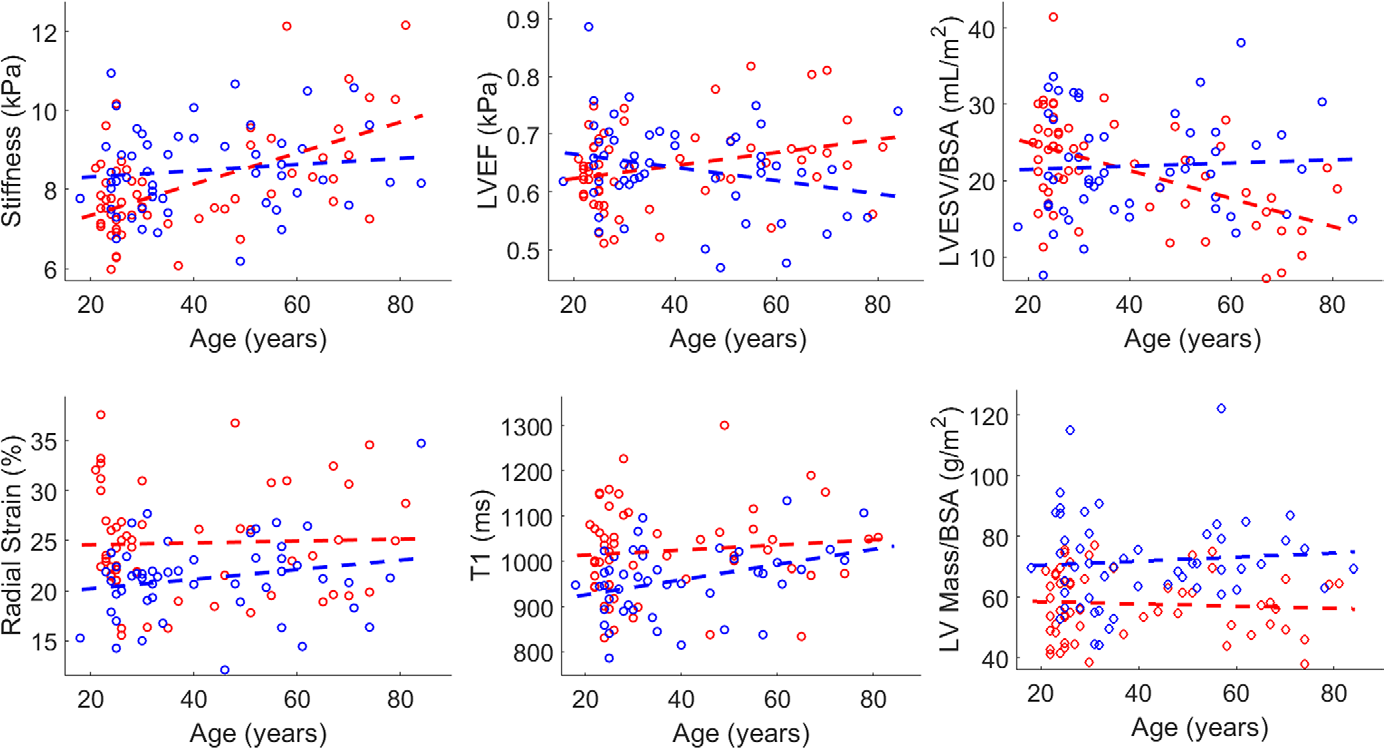
Cardiac MR elastography and cardiac MRI metrics as a function of age and sex. Red and blue circles depict female and male data points, respectively. The red and blue dotted lines depict the independent model fits to the female and male data, respectively. BSA = body surface area, LV = left ventricular, LVEF = LV ejection fraction, LVESV = LV end-systolic volume.

All outputs of the linear regression model are summarized in Table 2, and some significant correlations are shown in Figure 2. The intercept in Table 2 represents the predicted value, at the mean age of 40 years old, for the listed metric (dependent variable) when the age, sex, and age-sex interaction coefficients are set to zero. In the shear stiffness versus age plot in Figure 2, the trend lines for men (blue dashed line) and women (red dashed line) cross one another at the age of 51 years, which corresponds to the median age of menopause in the United States. Cardiac MRE–measured shear stiffness demonstrated an interaction with age and sex, where women experienced an increase in stiffness with age (age slope = 0.03 kPa/year ± 0.01, *P* = .009) and men (age slope = 0.008 kPa/year ± 0.009, *P* = .38) did not (Table 2). Age-sex interactions were also found for LV end-systolic volume (LVESV) over body surface area (BSA) and LVEF, where women experienced an increase in LVEF (age · sex slope = 0.0023 per year ± 0.0008, *P* = .005) and a decrease in LVESV/BSA (age · sex slope = −0.20 mL/m^2^ per year ± 0.07, *P* = .003). Native T1, circumferential/longitudinal/radial strain, and LV mass index, did not demonstrate an age-sex interaction (*P* > .05). However, women at the mean age of the sample (40 years ± 18) demonstrated higher radial strain (3.6% ± 0.9, *P* < .001) and T1 (65 msec ± 18, *P* < .001) and lower circumferential strain (−1.3% ± 0.4, *P* < .001), longitudinal strain (−1.6% ± 0.6, *P* = .007), and LV mass index (−14 g/m^2^ ± 3, *P* < .001) than men. The mean ± SD of OSS-SNR, a metric of MRE image quality, was 1.7 ± 0.4 across all participants and did not show any significant correlations with age, sex, or age-sex interactions. For reference purposes, the mean MRI/MRE metrics have been summarized in Table 3 for each decade. Again, an increase in myocardial shear stiffness can be observed in women as they transition from their 40s to their 50s.

**Table 2:**
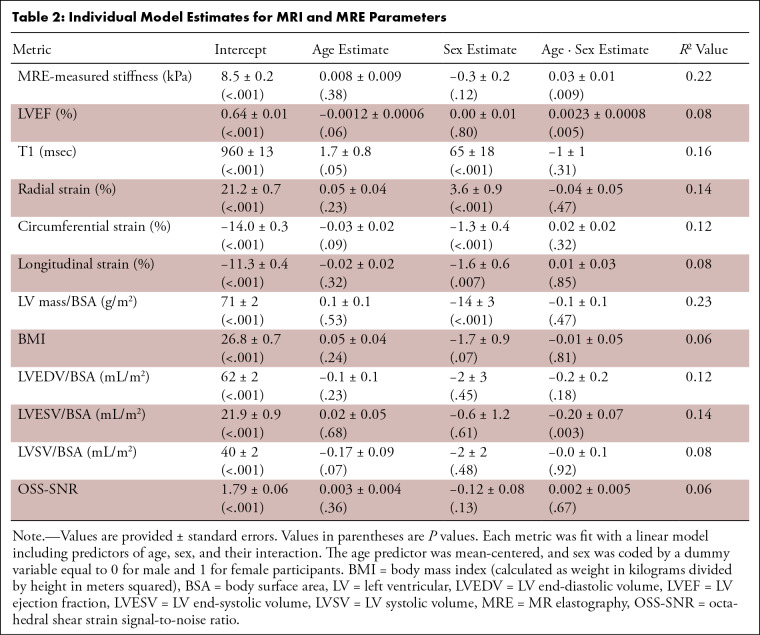
Individual Model Estimates for MRI and MRE Parameters

**Table 3:**
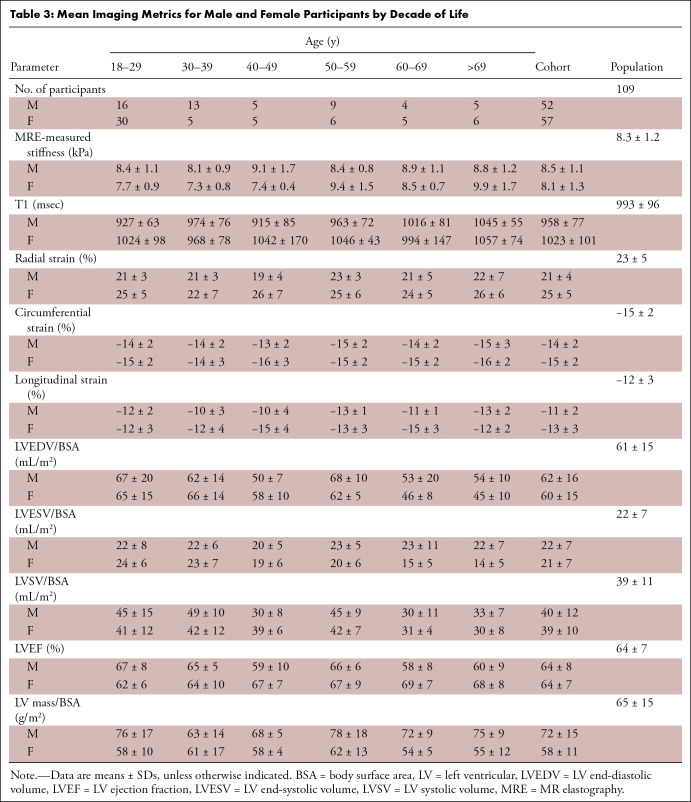
Mean Imaging Metrics for Male and Female Participants by Decade of Life

### Correlations between MRE and MRI and SNR Metrics

**Figure 3:**
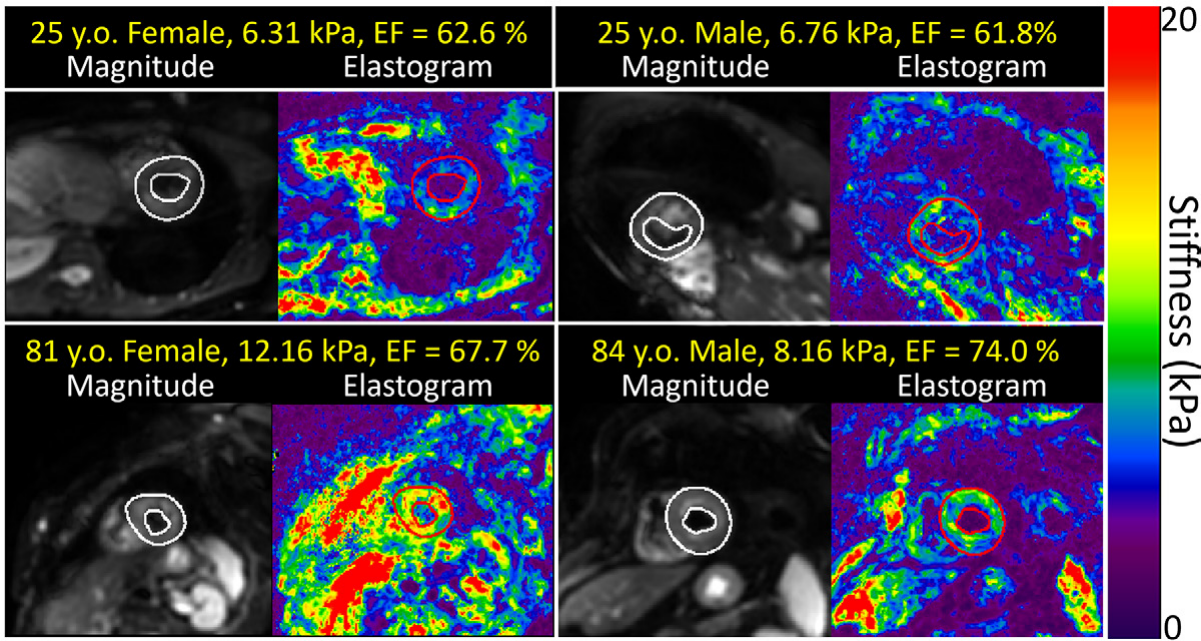
The range of short-axis left ventricular stiffness maps observed with MRE in younger and older healthy volunteers. The regions of interest used for the cardiac MRE measurements are outlined in white in the short-axis MRI magnitude images and red in each corresponding elastogram. The ejection fraction (EF) was not significantly correlated with myocardial stiffness. There was no use of contrast media for any images. MRE = MR elastography.

**Figure 4:**
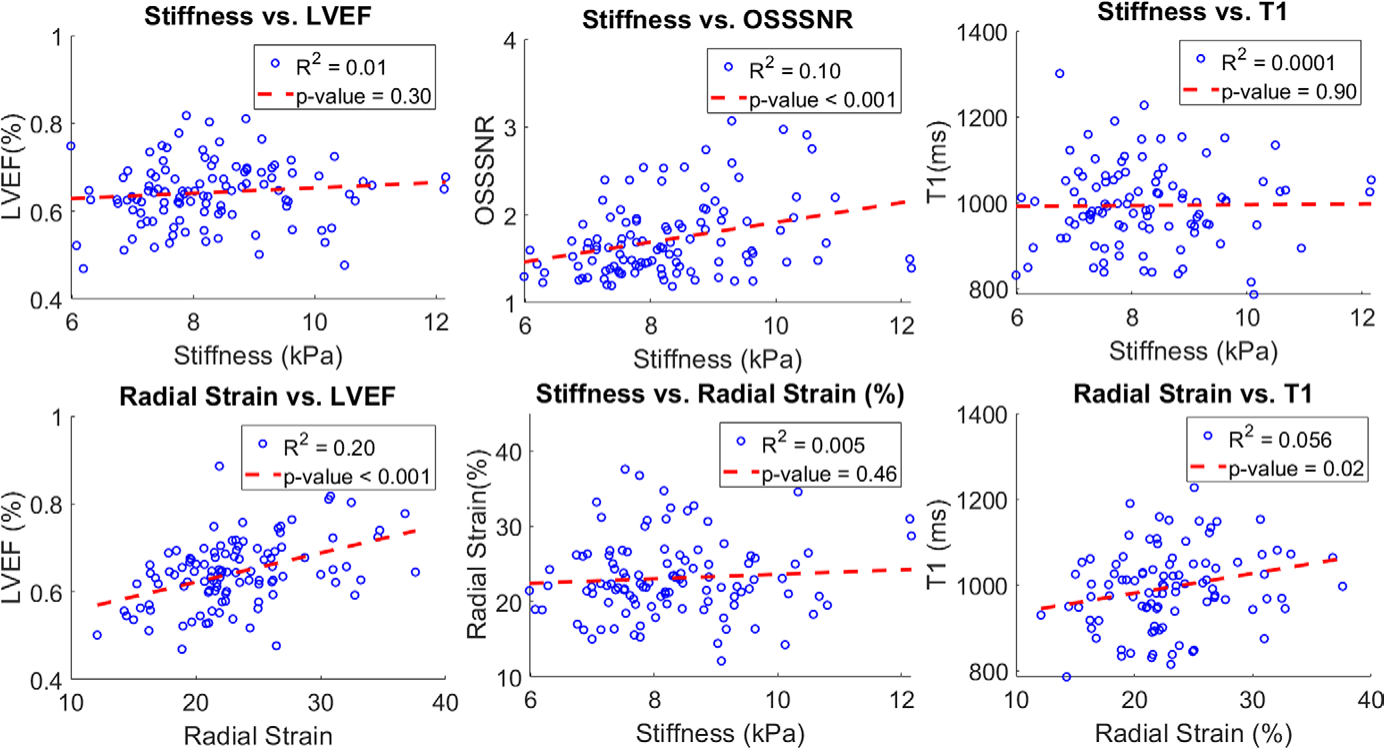
Comparison of T1, LVEF, and OSS-SNR as a function of cardiac MR elastography (MRE)–measured stiffness and radial strain. Cardiac MRE–measured stiffness was not correlated with T1, LVEF, or radial strain, while radial strain was correlated with both T1 and LVEF. This suggests that cardiac MRE–measured stiffness is a complementary measurement to strain and common MRI metrics. Stiffness estimates also showed a weak correlation with OSS-SNR, an estimate of wave quality. LVEF = left ventricular ejection fraction, OSS-SNR = octahedral shear strain signal-to-noise ratio.

Estimates of MRE-measured shear stiffness as a function of MRI and wave signal-to-noise (OSS-SNR) are summarized in Table 4. A full correlation and *P* value matrix for all metrics in Table 4 have been provided in Tables S1 and S2, respectively. MRE-measured shear stiffness correlated with LV end-diastolic volume/BSA (*r* = −0.28), LVESV/BSA (*r* = −0.28), and OSS-SNR (*r* = 0.31, weak correlation), but the correlation coefficients showed weak relationships (*r* < 0.4). On the other hand, longitudinal (*P* < .001, *r* = −0.37), radial (*P* < .001, *r* = 0.44), and circumferential (*P* < .001, −*r* = 0.44) strain were correlated with LVEF with a weak-moderate correlation. Examples of representative shear stiffness maps in a central short-axis LV section are shown in Figure 3, and linear regression plots of shear stiffness and some MRI metrics are shown in Figure 4. In the 16 participants with both reduced-field-of-view and full-field-of-view cardiac MRE scans, an intraclass correlation coefficient and concordance coefficient of 0.78 and 0.76, respectively, were calculated between the stiffness of both scans, indicating good reliability of cardiac MRE–measured shear stiffness.

**Table 4:**
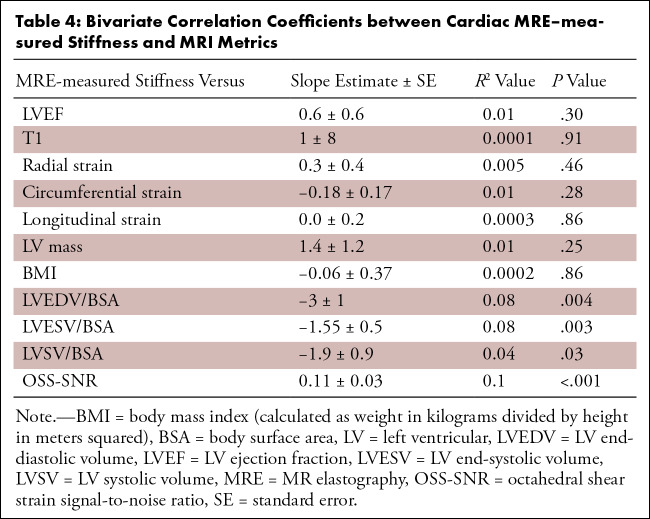
Bivariate Correlation Coefficients between Cardiac MRE–measured Stiffness and MRI Metrics

## Discussion

Our study demonstrated a significant increase in cardiac MRE–derived LV myocardial shear stiffness with age in women (*P* = .009) but not in men. LVEF increased (*P* = .005) and LVESV decreased (*P* = .003) disproportionately with age in women. Conventional MRI parameters, including native T1, circumferential/longitudinal/radial strain, and LV mass, did not demonstrate this interaction (*P* > .05). MRE-measured shear stiffness was not correlated with LVEF, body mass index, LV mass, or any MRI strain metrics (*P* > .05). To ensure that geometric variations did not significantly impact these findings, a secondary analysis found no correlation between MRE-measured shear stiffness (*P* = .21, *R*^2^ = 0.014) and the maximum LV thickness obtained from masks drawn directly on the MRE magnitude images (Fig S1). These data support the hypothesis that female individuals (but not male individuals) experience a disproportionate elevation in LV stiffness as they age and that MRE may be a complementary marker to existing MRI metrics.

These data also demonstrate that the hearts of female individuals are generally softer than those of male individuals at younger ages but transition to becoming stiffer around the median age of menopause (51 years of age). This suggests that the transition of myocardial stiffening may begin prior to the onset of menopause and progresses afterward. Several postulated mechanisms for elevated myocardial stiffness are associated with low estrogen levels, inducing elevated collagen synthesis and downregulating protein kinase A, which then influences the phosphorylation state of titin and the heart’s mechanical properties (24). Although this was not the objective of the current study, studying myocardial shear stiffness during the menopausal transitional period could help identify cardiac MRE as a valuable early biomarker for heart failure.

To date, a majority of cardiac MRE studies have focused on demonstrating changes in myocardial shear stiffness in disease states (17,25–27), different phases in the cardiac cycle (3), and anisotropy (26). The interaction between LV MRE-measured shear stiffness and aging was initially investigated by Wassenaar et al (28), at an 80-Hz vibration frequency, using a two-dimensional MRE approach, and in 29 healthy volunteers (11 female). They reported a weak linear correlation between shear stiffness and age but did not report any sex differences. However, the authors did report a similar concordance correlation coefficient (0.77) that is in good agreement with our intraclass correlation coefficient (0.78) and concordance coefficient (0.76) findings.

Elgeti et al (29) used an alternative shear wave amplitude measurement MRE technique (30) to distinguish patients with diastolic abnormalities at echocardiography from healthy individuals. However, their technique did not acquire 3D displacement fields, and there was no attempt to quantitate myocardial shear stiffness. Instead, the authors reported the mean amplitudes of the shear wave displacement as a surrogate for myocardial shear stiffness (29). In their 2010 article (4), the authors showed that younger volunteers (25–35 years, *n* = 10) had a higher LV amplitude to reference amplitude ratio than older volunteers (50–60 years, *n* = 5), and both volunteer groups had significantly higher ratios than patients (44–77 years, *n* = 10) with proven relaxation abnormalities. These results support the hypothesis that myocardial shear stiffness increases with age and disease, but again, no sex interaction was evaluated.

The cardiac US elastography literature has also focused on demonstrating feasibility in healthy adults and pediatric volunteers (31,32). Using shear wave imaging with US, Song et al (32) showed that 20 children in the age range of 5–18 years did not show any sex differences or aging-associated changes with myocardial shear stiffness. This same group also did a feasibility study in 10 healthy volunteers (nine men) in the age range of 23–61 years but could not perform age-associated and sex-stratified analysis due to small sample sizes. More recently, Petrescu et al (33) demonstrated that intrinsic mitral and aortic valve closure-induced myocardial shear wave velocities increased with age in 50 healthy volunteers (20–80 years old), but they did not investigate sex differences. It should also be noted that intrinsic velocity estimates are going to be more strongly impacted by waveguide/geometric effects as well as wave dispersion effects and contribute to differences in shear stiffness estimates when compared with the steady-state single-frequency, 3D MRE technique reported here. Nevertheless, these studies support the hypothesis that myocardial stiffness increases with age, but due to the lack of sex-stratified analysis, it is unclear if the primary contributors to this trend are female individuals, as observed in our current study. This highlights the need for the inclusion of sex-stratified analyses in myocardial stiffness studies.

One of the most important findings of this current study is that the point of intersection, where the myocardial shear stiffness of female participants becomes higher than that of male participants, is at the age of 51 years, the median age of menopause in the United States. Interestingly, although myocardial shear stiffness increased in older female participants, two commonly accepted biomarkers, LVEF and LVESV/BSA, showed improvement in these participants. Furthermore, the lack of correlation between cardiac MRE–measured shear stiffness estimates with LVEF, as well as strain metrics, suggests that MRE gives complementary information to existing estimates of myocardial function and contractility. If myocardial stiffening is associated with a negative prognosis, the potential mechanisms for its discordance with LVEF and LVESV/BSA will need to be investigated in future studies. It must be noted that this current study only investigated changes in healthy participants with no known cardiovascular disease, while studies that have shown associations between myocardial stiffness, as defined by pressure-volume loops, and metrics such as native T1, extracellular volume fractions, or contractility metrics, are conducted in participants with known heart disease (34–36). Therefore, a discordance between MRE-measured shear stiffness with existing diagnostic markers in healthy volunteers does not mean that a trend does not exist once patient cohorts are investigated. Nevertheless, it is likely that menopausal transition in female individuals will become a unique window of opportunity to understand such mechanisms and administer and monitor new and existing therapies, which could substantially help improve their long-term health.

This study had limitations, as it was an early attempt at performing 3D high-frequency cardiac MRE in a sample of healthy volunteers. First, the wave inversion used to generate shear stiffness maps in this study assumed tissue isotropy and local homogeneity, which are not true in the heart and may introduce errors in the stiffness measurement accuracy. In addition, although attempts were made to ensure image quality was consistent and above a certain threshold using an OSS-SNR metric, SNR variations between participants could still have an impact on stiffness estimates. In future studies, it may be beneficial to account for anisotropy (5,37) to obtain the full stiffness tensor (38), improve inversion algorithms to make them more robust to noise, and potentially improve the effectiveness of the curl operator for stiffness calculation (39,40). Second, a 5-mm isotropic acquisition resolution was used, which provided suboptimal spatial resolution and limited interrogation of tissue waves across the myocardium but was necessary to ensure high SNR and good shear wave detectability. Third, only an early systolic phase was investigated with MRE in this study, as this was the most reproducible phase and gave more reliable shear stiffness estimates due to the thicker myocardium. Measuring multiple phases in the cardiac cycle, particularly end diastole, will be desirable in the future for the evaluation of restrictive myocardial diseases, which are thought to predominantly affect diastole. In addition, due to the limited 25-mm superior-inferior coverage of the heart volume, only global measurements were reported. Future studies looking at changes in localized myocardial stiffness in different volunteer and patient populations could be more informative of the cause of myocardial stiffening. Fourth, although no incidental findings were observed at the clinical MRI examinations, these healthy volunteers did not have a full clinical workup (ECG, echocardiography, or stress test) or a full history (smoking, pre-existing conditions, etc) performed, which could leave opportunity for undetected cardiovascular disease. Nevertheless, the outcomes of this study motivate the investigation of cardiac MRE in female individuals transitioning through menopause and heart failure populations known to be impacted by myocardial stiffness. Last, because this was a volunteer population, clinical indicators from blood tests or even blood pressure measurements were not available. However, previous studies involving ex vivo myocardial mechanical testing have demonstrated that stiffness is more dominantly impacted by titin and collagen depositions than by hypertension alone (41).

In conclusion, our study demonstrates that female but not male individuals experience a disproportionate elevation in LV shear stiffness measured with cardiac MRE as they age and that MRE may be a complementary marker to existing MRI metrics. Future studies evaluating the impact of menopause transition on myocardial shear stiffness and the associations with heart failure are needed. A better understanding of these sex-related differences and their association with aging may help reduce sex disparities in individuals with heart failure.
